# Causal effects of human serum metabolites on occurrence and progress indicators of chronic kidney disease: a two-sample Mendelian randomization study

**DOI:** 10.3389/fnut.2023.1274078

**Published:** 2024-01-08

**Authors:** Yu Yin, Conghui Shan, Qianguang Han, Congcong Chen, Zijie Wang, Zhengkai Huang, Hao Chen, Li Sun, Shuang Fei, Jun Tao, Zhijian Han, Ruoyun Tan, Min Gu, Xiaobing Ju

**Affiliations:** ^1^Department of Urology, The First Affiliated Hospital of Nanjing Medical University, Nanjing, China; ^2^Department of Clinical Laboratory, The First Affiliated Hospital of Nanjing Medical University, Nanjing, China; ^3^Department of Urology, The Second Affiliated Hospital of Nanjing Medical University, Nanjing, China

**Keywords:** chronic kidney diseases (CKD), metabolites, two-sample Mendelian randomization (two-sample MR), mannose, porphyrin metabolism

## Abstract

**Background:**

Chronic kidney disease (CKD) is often accompanied by alterations in the metabolic profile of the body, yet the causative role of these metabolic changes in the onset of CKD remains a subject of ongoing debate. This study investigates the causative links between metabolites and CKD by leveraging the results of genomewide association study (GWAS) from 486 blood metabolites, employing bulk two-sample Mendelian randomization (MR) analyses. Building on the metabolites that exhibit a causal relationship with CKD, we delve deeper using enrichment analysis to identify the metabolic pathways that may contribute to the development and progression of CKD.

**Methods:**

In conducting the Mendelian randomization analysis, we treated the GWAS data for 486 metabolic traits as exposure variables while using GWAS data for estimated glomerular filtration rate based on serum creatinine (eGFRcrea), microalbuminuria, and the urinary albumin-to-creatinine ratio (UACR) sourced from the CKDGen consortium as the outcome variables. Inverse-variance weighting (IVW) analysis was used to identify metabolites with a causal relationship to outcome. Using Bonferroni correction, metabolites with more robust causal relationships are screened. Additionally, the IVW-positive results were supplemented with the weighted median, MR-Egger, weighted mode, and simple mode. Furthermore, we performed sensitivity analyses using the Cochran Q test, MR-Egger intercept test, MR-PRESSO, and leave-one-out (LOO) test. Pathway enrichment analysis was conducted using two databases, KEGG and SMPDB, for eligible metabolites.

**Results:**

During the batch Mendelian randomization (MR) analyses, upon completion of the inverse-variance weighted (IVW) approach, sensitivity analysis, and directional consistency checks, 78 metabolites were found to meet the criteria. The following four metabolites satisfy Bonferroni correction: mannose, N-acetylornithine, glycine, and bilirubin (Z, Z), and mannose is causally related to all outcomes of CKD. By pathway enrichment analysis, we identified eight metabolic pathways that contribute to CKD occurrence and progression.

**Conclusion:**

Based on the present analysis, mannose met Bonferroni correction and had causal associations with CKD, eGFRcrea, microalbuminuria, and UACR. As a potential target for CKD diagnosis and treatment, mannose is believed to play an important role in the occurrence and development of CKD.

## 1 Introduction

Chronic kidney disease (CKD) results from a variety of etiologies, characterized by reduced renal function as a clinical manifestation and renal fibrosis as a pathologic feature (1, 2). According to the latest KDIGO Guidelines, the most widely recognized indicator of kidney damage is the marker of kidney damage or GFR > 60 mL/min/1.73 m^2^ for >3 months (3). According to the most recent epidemiological study on CKD on a global scale, approximately 700 million people suffer from CKD worldwide as of 2017 (4). Around the world, CKD has increased in incidence and mortality in recent years (5, 6), and CKD is predicted to become the fifth leading cause of death worldwide by 2040 (7). As CKD progresses, kidney function will gradually and irreversibly decline. CKD progresses to kidney failure when the glomerular filtration rate [GFR] < 15 mL/min/1.73 m^2^ (8). In the stage of kidney failure, there are only two treatment options left: dialysis and kidney transplantation (9). In a previous study of 228,552 patients with kidney failure, based on standardized mortality, kidney transplantation was associated with a survival advantage over dialysis (10). Patients who undergo surgery or under dialysis face a substantial financial burden as well as risks associated with the procedure (11). Therefore, early identification of risk factors and prevention of CKD are particularly important.

Metabolomics is a research method that analyzes metabolites (usually small molecules with a molecular weight < 1,000 Daltons) produced by various biological processes in a target sample (12). Levels of metabolite products are genetically determined and interact with environmental factors to ultimately create differences between individuals (13, 14). Patients with CKD, especially those who progress to kidney failure, are affected by the disease itself and subsequent interventions and suffer from metabolic disturbances, such as elevated lipids, abnormally hypercatabolic catabolism, and iron metabolism disorder (15–17). In recent years, more and more researchers have tried to explore the mechanisms of CKD progression and new diagnostic and therapeutic targets through the study of metabolites in patients’ biospecimens (18–20).

Using whole genome sequencing data, Mendelian randomization (MR) can be used to examine the causal relationship between exposure and outcome in disease etiology studies (21). This research method using innate genotype as the instrumental variable (IVs), imitating the RCT study in methodology, makes the final conclusion more reliable and greatly reduces the possibility of reverse causality (22, 23). Because of the above advantages of MR Analysis, more and more researchers are choosing to explore the etiology of diseases based on previous GWAS results. Based on the MR analysis, Ponte et al. investigated the causal relationship between uromodulin, blood pressure, and chronic kidney diseases (24). MR analysis by Kjaergaard et al. found that obesity affects kidney function and proteinuria caused by diabetes type 2 (25). With the establishment of an atlas of genetically determined metabotypes (GDMs) (26), we can investigate the causal relationship between blood metabolites and CKD through MR analysis.

In this study, as exposure factors, we used 486 blood metabolites, and CKD as well as its related test indicators as outcome factors, and then, MR analysis was conducted to determine which metabolites were causally associated with CKD. In order to identify the pathways that may cause CKD, an enrichment analysis of metabolic pathways was performed based on the positive results of the MR analysis. The results of this study provide a new target and direction for the screening of future CKD patients as well as early intervention and even treatment to delay the progression of the disease.

## 2 Materials and methods

### 2.1 Study design and data source

The MR analyses we perform follow the three assumptions underlying MR: (1) There is a strong association between IVs and exposures; (2) IVs are independent of confounding factors; (3) IVs affect outcomes only via exposures and are not directly related to outcome; the specific hypotheses regarding the MR analysis of this study are detailed in Supplementary Figure 1, while the overview of the research process for this study can be found in the flowchart (Supplementary Figure 2).

A previous GWAS analysis of 486 metabolites in human blood was used to assess exposure in this study (26), and then, we investigated the consequential impacts of each metabolite on CKD and CKD-related indicators using two-sample MR. A total of 7,824 Europeans were included in the metabolite GWAS study, including 1768 from the KORA F4 study in Germany and 6,056 from the UK Twin Study; specific GWAS results can be downloaded at metabolomics GWAS server.^1^ Apart from 107 metabolites of unknown function, the remaining 486 metabolites were organized into eight groups, namely: amino acid, carbohydrate, cofactors and vitamin, energy, lipid, nucleotide, peptide, and xenobiotic metabolism.

GWAS results for CKD, eGFR based on serum creatinine (eGFRcrea), microalbuminuria, and urinary albumin-to-creatinine ratio (UACR) obtained from the CKDGen Consortium, downloaded from the IEU OpenGWAS project,^2^ which are all European-ancestry samples. CKD and eGFRcrea were derived from the previous studies of Pattaro et al. (27). There were 117,165 samples in the CKD study (ncase = 12,385; ncontrol = 104,780) and 133,814 samples in the eGFRcrea study. Microalbuminuria and UACR come from previous studies by Teumer et al. (28). The study of UACR contains 54,450 samples, and the study of microalbuminuria contains 54,116 samples.

### 2.2 The selection of instrumental variables

Considering that the number of metabolite-related SNPs in the blood is not optimistic, we chose *p* < 10^−5^ as the screening criteria for strongly associated IVs with exposure factors. We then set the linkage disequilibrium parameter (R2 > 0.1 and within 500 kb) to ensure that the IVs are independent. Our next step was to eliminate weaker IVs by using *F* > 10 as the standard, and the specific *F* value was obtained using the following formula:


$$R^{2}=\frac{2*\beta^{2}*EAF*\left(1-EAF\right)}{\left[2*\beta^{2}*EAF*\left(1-EAF\right)+2*{\left(se\;\left(\;\beta \right)\right)}^{2}*N*EAF*\left(1-EAF\right)\right]}$$

$$F=\frac{N-k-1}{k}*\;\frac{R^{2}}{1-R^{2}}$$

Previous similar studies have explained the specific formula (29). Following removal of the IVs directly associated with the outcome (*p* = 1 × 10 5), we queried the IVs associated with confounders (hypertension, diabetes, kidney disease, and obesity) with Phenoscann. The harmonise_data function in the TwoSampleMR package is then used to perform data harmonization to ensure that the effect allele belongs to the same allele. Finally, we conducted MR analysis between metabolites with IVs greater than two and our outcome time.

### 2.3 MR analysis

All data analysis was based on R (version 4.1.1). First, based on the inverse-variance weighted (IVW) method, the Wald ratio of IVs was systematically evaluated using *p* < 0.05 as the screening condition. The TwoSampleMR package (version 0.5.7) was used to screen and analyze metabolites associated with CKD and its related indicators. Further analyses were conducted only on metabolites whose results were in the same direction as those obtained by all five methods (inverse-variance weighted, weighted median, MR-Egger, weighted mode, and simple mode). In order to evaluate heterogeneity and pleiotropy of IVs, Cochran’s Q test and MR-Egger intercept analysis were conducted, respectively, and those metabolites that passed the IVW test but had pleiotropy (*p* < 0.05) were excluded. Utilize MR-PRESSO (version 1.0) to identify and remove outlier SNP in IVs. By using the leave-one-out (LOO) analysis, we ensured that MR Results would not be impacted by any single SNP. The above analysis was conducted with CKD, eGFRcrea, UACR, and microalbuminuria as the outcomes, and then, we intersected the eligible results to examine the core metabolites contributing to CKD progression. Bonferroni correction was used to identify metabolites with obvious causal relationships, as the process of metabolite screening involved multiple MR analyses.

### 2.4 Enrichment analysis of metabolic pathway

The enrichment analysis of metabolic pathways was applied to the metabolites that have causal relationship with CKD, eGFRcrea, UACR, and microalbuminuria, which meet all the conditions for the above MR analysis, using MetaboAnalyst 5.0.^3^ To further explore the metabolic pathways associated with CKD, two databases were employed, KEGG and SMPDB.

### 2.5 Validation of MR results

To further validate our conclusions, we used the new GWAS database for validation. First, we extracted GWAS data for metabolites that met the Bonferroni correction in the MR results from a GWAS library containing 1,091 metabolites and 309 metabolite ratios as a validation of the exposure to MR analysis. We then used the GWAS results with study accession ID GCST008059 in the catalog database as a validation of the end of the MR analysis. The study included GWAS data of eGFR with 567,460 European-ancestry individuals. The criteria and steps for screening subsequent IVs were exactly the same as before.

## 3 Result

### 3.1 Preparation of IVs

In a series of screenings with a strong correlation to a metabolite but no correlation to CKD, LD analysis, and screening with *F* > 10, 8,167 SNPS met the criteria. As a result of searching Phenoscann, screening and deleting SNPS strongly associated with confounders, Supplementary Table 1 contained 8,147 SNPS for further MR analysis. Supplementary Table 2 shows SNPS associated with confounders and deleted. Finally, we removed blood metabolites with IVs less than three for subsequent MR analysis.

### 3.2 MR analysis

**Figure 1:**
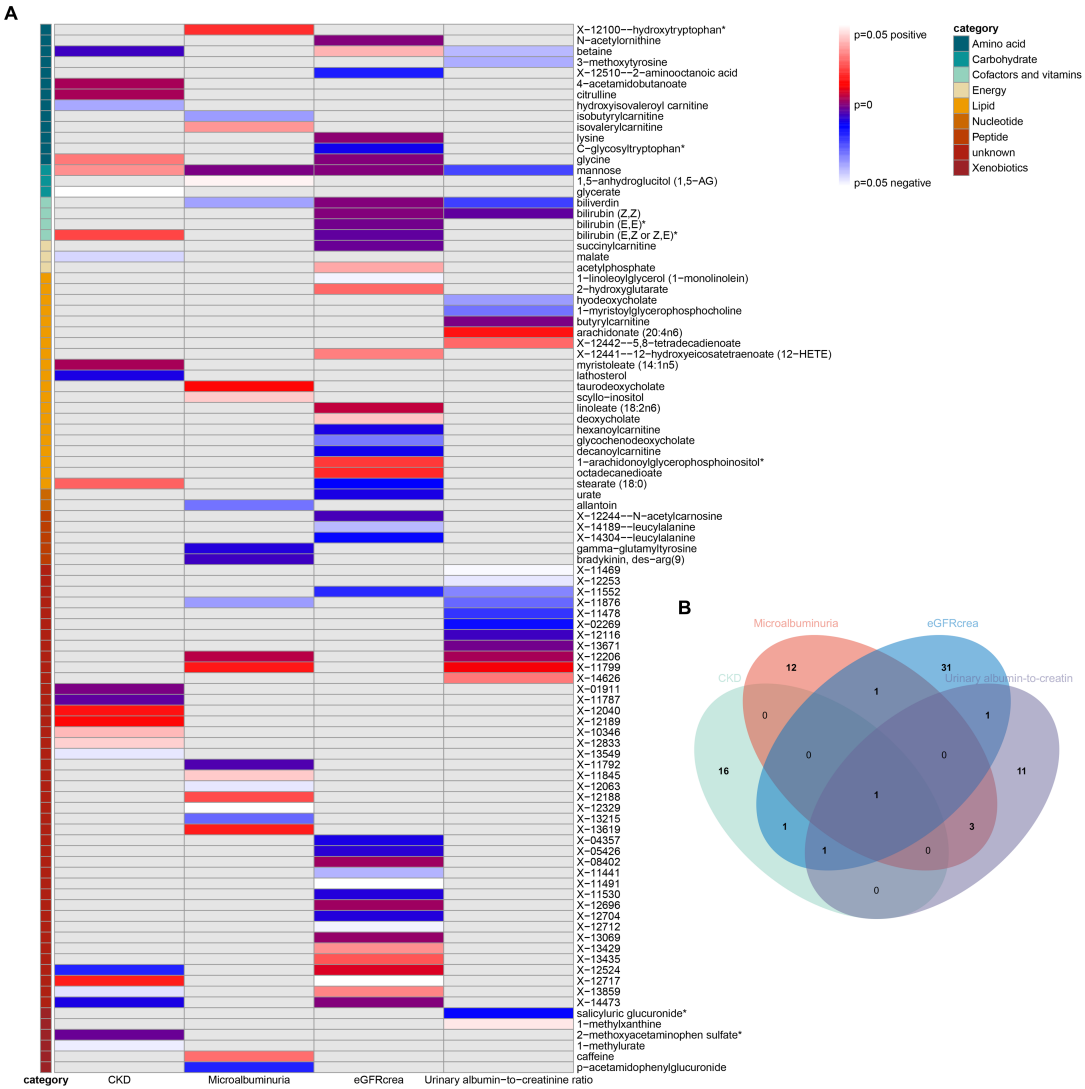
Heatmaps and Venn diagram visualizing IVW analysis results for CKD, eGFRcrea, microalbuminuria, and UACR. **(A)** Heatmaps showing the *p*-values of the IVW analysis and the direction of the result. **(B)** Venn diagram showing the intersection of all positive MR results. CKD, Chronic Kidney Disease; eGFRcrea, eGFR based on serum creatinine; UACR, urinary albumin-to-creatinine ratio.

**Figure 2:**
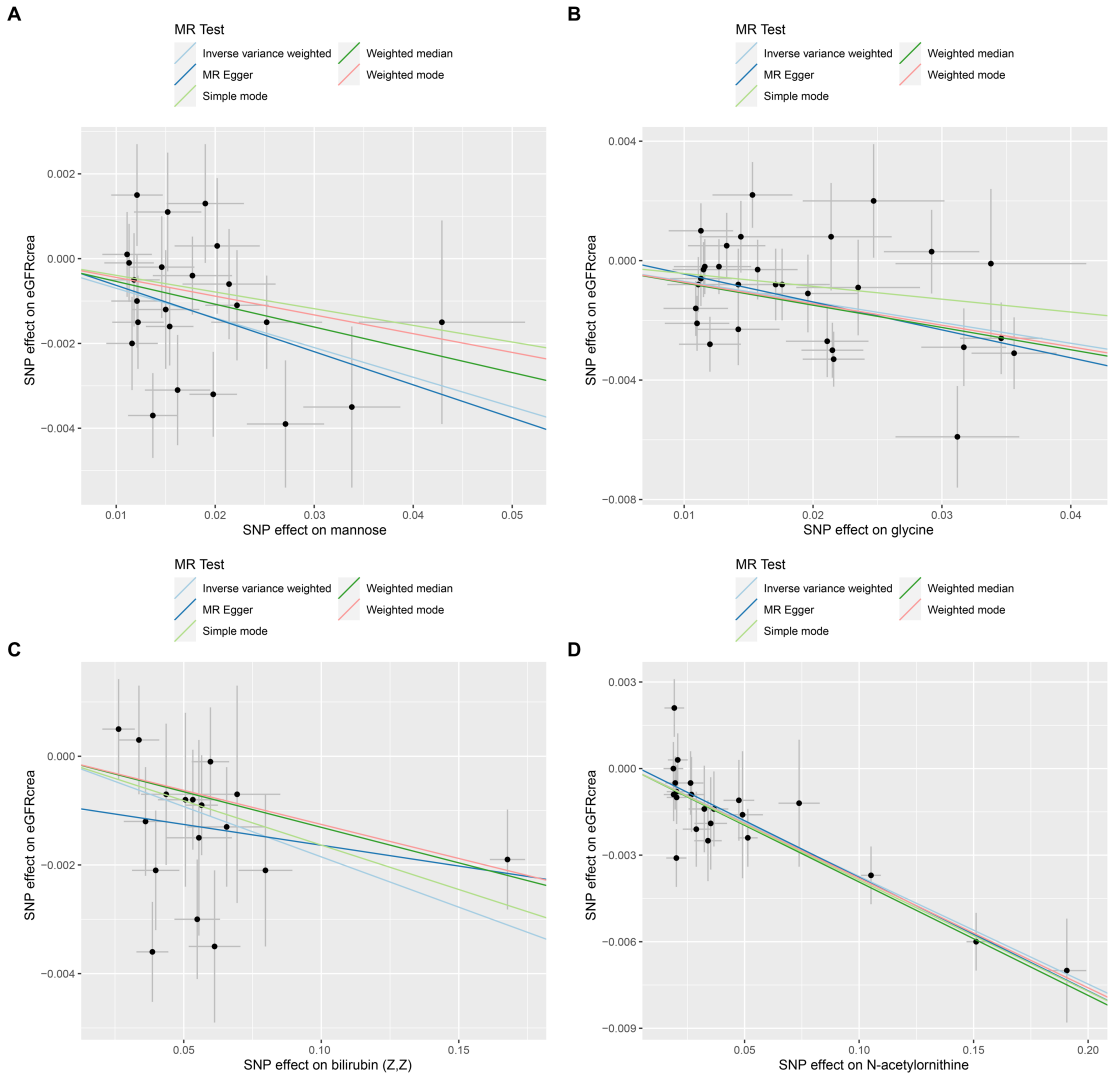
Scatter plots of the four metabolites that conform to the Bonferroni correction show causal relationships with eGFRcrea. **(A)** Mannose, **(B)** glycine, **(C)** bilirubin (Z, Z), **(D)** N-acetylornithine.

**Figure 3:**
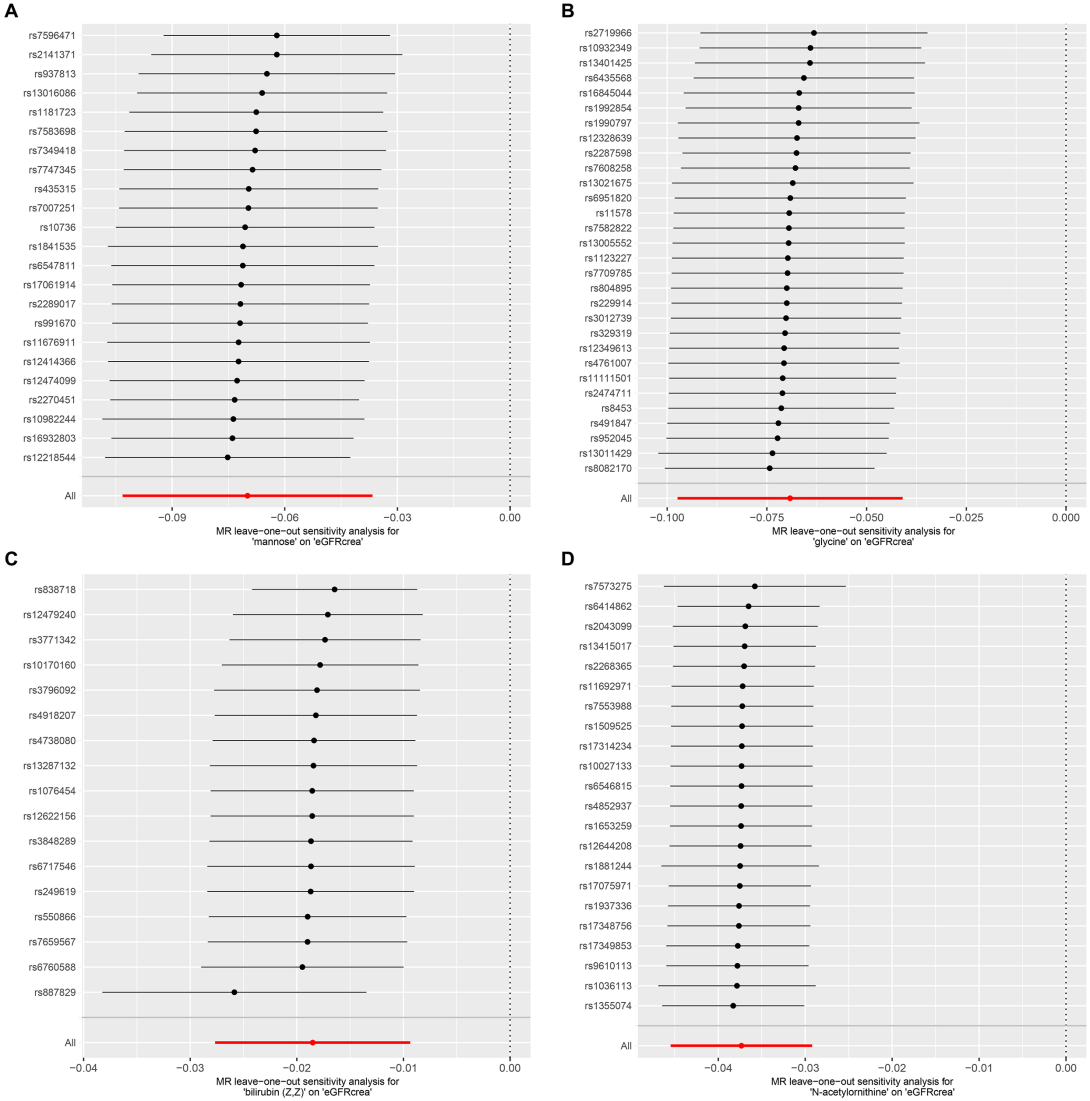
LOO analysis of four metabolites conforming to the Bonferroni correction. **(A)** Mannose, **(B)** glycine, **(C)** bilirubin (Z, Z), **(D)** N-acetylornithine. LOO analysis: leave-one-out analysis.

As the first step, we screened metabolites with *p*-values less than 0.05 in IVW analysis with CKD, eGFRcrea, microalbuminuria, and UACR as outcomes. In total, 116 metabolites were investigated, including 62 known substances and 54 unknown substances, which were visualized as heatmaps (Figure 1A). One hundred sixteen metabolites were then analyzed by four subsequent MR analyses (weighted median, MR-Egger, weighted mode, and simple mode). Supplementary Table 3 shows metabolites in the same direction as the screening results from the five methods. Seventy-eight metabolites that met the criteria, and their IVW analysis results are listed in Table 1, as well as their heterogeneity and pleiotropy test results in Supplementary Table 4. Following that, we intersected the four outcomes and drew the Venn diagram (Figure 1B). In addition to being causally related to all four outcomes, mannose is a risk factor for CKD, while being negatively correlated with the increase in eGFRcrea, UACR, and microalbuminuria. Four metabolites that are eligible for analysis after Bonferroni correction: mannose, N-acetylornithine, glycine, and bilirubin (Z, Z). This scatterplot shows the effects of these four metabolites on eGFRcrea changes in five different MR methods (Figure 2). Based on the LOO method (Figure 3), no single SNP would impact the MR Results of the four metabolites tested.

**Table 1:** The IVW results of metabolites were consistent with the sensitivity analysis in the same direction of the five MR analysis results.

	CKD		Microalbuminuria		eGFRcrea		Urinary albumin-to-creatinine ratio	
	OR(95%CI)	*P*	OR(95%CI)	*P*	OR(95%CI)	*P*	OR(95%CI)	*P*
Malate	0.45(0.21,0.98)	0.0438	–	–	–	–	–	–
4-acetamidobutanoate	2.16(1.29,3.63)	0.0035	–	–	–	–	–	–
Glycerate	0.46(0.22,1)	0.0496	–	–	–	–	–	–
Citrulline	2.3(1.32,4.01)	0.0031	–	–	–	–	–	–
Myristoleate (14:1n5)	2.04(1.26,3.29)	0.0036	–	–	–	–	–	–
X-01911	0.64(0.5,0.83)	0.0005	–	–	–	–	–	–
X-11787	0.4(0.21,0.74)	0.0036	–	–	–	–	–	–
2-methoxyacetaminophen sulfate	0.96(0.94,0.99)	0.0027	–	–	–	–	–	–
X-12040	1.11(1.02,1.21)	0.0153	–	–	–	–	–	–
Lathosterol	0.67(0.5,0.91)	0.0095	–	–	–	–	–	–
X-12189	1.13(1.02,1.24)	0.0134	–	–	–	–	–	–
X-10346	1.22(1.01,1.48)	0.0389	–	–	–	–	–	–
X-12524	0.3(0.11,0.82)	0.0185	–	–	1.09(1.02,1.16)	0.0081	–	–
X-12833	1.1(1,1.22)	0.0422	–	–	–	–	–	–
X-13549	0.38(0.15,0.98)	0.0459	–	–	–	–	–	–
Hydroxyisovaleroyl carnitine	0.44(0.21,0.95)	0.037	–	–	–	–	–	–
Caffeine	–	–	1.42(1.04,1.93)	0.0279	–	–	–	–
Allantoin	–	–	0.65(0.43,0.96)	0.0297	–	–	–	–
Gamma-glutamyltyrosine	–	–	0.4(0.2,0.8)	0.0093	–	–	–	–
Scyllo-Inositol	–	–	1.94(1.03,3.67)	0.0415	–	–	–	–
X-11792	–	–	0.73(0.59,0.91)	0.0046	–	–	–	–
X-11845	–	–	1.23(1.01,1.49)	0.0414	–	–	–	–
X-12063	–	–	0.82(0.68,1)	0.0458	–	–	–	–
p-acetamidophenylglucuronide	–	–	0.96(0.93,0.99)	0.0181	–	–	–	–
Isobutyrylcarnitine	–	–	0.67(0.46,0.97)	0.0354	–	–	–	–
X-12100–hydroxytryptophan	–	–	2.36(1.15,4.81)	0.0189	–	–	–	–
X-12188	–	–	1.23(1.03,1.46)	0.023	–	–	–	–
Bradykinin, des-arg(9)	–	–	0.81(0.7,0.94)	0.0066	–	–	–	–
Stearate (18:0)	1.91(1.08,3.39)	0.0264	–	–	0.95(0.92,0.99)	0.0129	–	–
Urate	–	–	–	–	0.93(0.89,0.98)	0.0095	–	–
Acetylphosphate	–	–	–	–	1.07(1,1.14)	0.0365	–	–
N-acetylornithine	–	–	–	–	0.96(0.96,0.97)	2.54E-19	–	–
X-04357	–	–	–	–	0.95(0.91,0.99)	0.0099	–	–
X-05426	–	–	–	–	0.97(0.94,0.99)	0.0082	–	–
X-08402	–	–	–	–	1.03(1.01,1.06)	0.0028	–	–
1-linoleoylglycerol (1-monolinolein)	–	–	–	–	0.98(0.97,1)	0.0479	–	–
Hexanoylcarnitine	–	–	–	–	0.98(0.96,0.99)	0.0096	–	–
Glycochenodeoxycholate	–	–	–	–	0.99(0.98,1)	0.0303	–	–
Bilirubin (E,E)	–	–	–	–	0.98(0.97,0.99)	0.0014	–	–
C-glycosyltryptophan	–	–	–	–	0.92(0.86,0.98)	0.0103	–	–
X-11491	–	–	–	–	1.01(1,1.03)	0.0491	–	–
X-11530	–	–	–	–	0.98(0.96,0.99)	0.0094	–	–
X-11552	–	–	–	–	0.98(0.96,1)	0.0191	0.84(0.71,0.99)	0.0324
X-12244–N-acetylcarnosine	–	–	–	–	0.95(0.92,0.99)	0.0061	–	–
X-12441–12-hydroxyeicosatetraenoate (12-HETE)	–	–	–	–	1.01(1,1.02)	0.0305	–	–
Bilirubin (E,Z or Z,E)	1.31(1.04,1.65)	0.0225	–	–	0.98(0.97,0.99)	0.0031	–	–
1-arachidonoylglycerophosphoinositol	–	–	–	–	1.03(1,1.06)	0.021	–	–
X-12696	–	–	–	–	1.03(1.01,1.06)	0.0025	–	–
X-12704	–	–	–	–	0.97(0.96,0.99)	0.0088	–	–
X-12712	–	–	–	–	0.99(0.99,1)	0.0476	–	–
X-13069	–	–	–	–	1.03(1.01,1.06)	0.002	–	–
X-13429	–	–	–	–	1.01(1,1.02)	0.0334	–	–
X-13435	–	–	–	–	1.03(1,1.06)	0.0245	–	–
X-13859	0.66(0.44,0.99)	0.0455	–	–	1.03(1,1.05)	0.0313	–	–
X-14189–leucylalanine	–	–	–	–	0.99(0.97,1)	0.0393	–	–
X-14304–leucylalanine	–	–	–	–	0.98(0.97,1)	0.0142	–	–
X-14473	0.62(0.43,0.89)	0.0096	–	–	1.04(1.02,1.06)	0.0002	–	–
Octadecanedioate	–	–	–	–	1.03(1.01,1.06)	0.0184	–	–
2-hydroxyglutarate	–	–	–	–	1.04(1.01,1.09)	0.0269	–	–
Arachidonate (20:4n6)	–	–	–	–	–	–	1.34(1.06,1.7)	0.0149
X-02269	–	–	–	–	–	–	0.88(0.79,0.98)	0.0149
X-11469	–	–	–	–	–	–	0.91(0.83,1)	0.0483
X-11478	–	–	–	–	–	–	0.83(0.7,0.97)	0.0203
Salicyluric glucuronide	–	–	–	–	–	–	0.93(0.87,0.98)	0.0136
X-12116	–	–	–	–	–	–	0.81(0.69,0.94)	0.0062
X-12253	–	–	–	–	–	–	0.84(0.71,1)	0.0457
X-12442–5,8-tetradecadienoate	–	–	–	–	–	–	1.18(1.02,1.36)	0.0272
1-methylxanthine	–	–	–	–	–	–	1.12(1,1.26)	0.0451
X-13671	–	–	–	–	–	–	0.64(0.49,0.85)	0.0016
1-myristoylglycerophosphocholine	–	–	–	–	–	–	0.76(0.59,0.97)	0.0297
Glycine	1.76(1.06,2.94)	0.0297	–	–	0.93(0.91,0.96)	1.55E-06	–	–
Biliverdin	–	–	0.76(0.59,0.98)	0.0362	0.98(0.96,0.99)	0.0003	0.91(0.83,0.99)	0.022
X-11799	–	–	1.38(1.06,1.8)	0.0156	–	–	1.11(1.02,1.2)	0.0109
X-11876	–	–	0.56(0.33,0.96)	0.0356	–	–	0.83(0.7,0.98)	0.0283
X-12206	–	–	3.37(1.45,7.83)	0.0048	–	–	1.58(1.17,2.13)	0.003
Bilirubin (Z,Z)	–	–	–	–	0.98(0.97,0.99)	0.0001	0.92(0.87,0.97)	0.0035
Betaine	0.48(0.28,0.81)	0.0062	–	–	1.05(1,1.1)	0.0377	0.78(0.62,0.99)	0.0396
Mannose	1.81(1.05,3.11)	0.0329	0.3(0.15,0.6)	0.0007	0.93(0.9,0.96)	3.84E-05	0.79(0.64,0.97)	0.0228

### 3.3 Analysis of metabolic pathway enrichment

The results of MR Analysis were enriched with the KEGG and SMPDB databases, resulting in eight meaningful pathways in Table 2. Caffeine metabolism, glycine serine and threonine metabolism, glycine and serine metabolism, glyoxylate and dicarboxylate metabolism, methionine metabolism, porphyrin and chlorophyll metabolism, porphyrin metabolism, and primary bile acid biosynthesis may be potential factors leading to the occurrence and even progression of CKD.

**Table 2:** Results of pathway enrichment analysis.

Metabolic Pathway	Trait	Database	*P*
Caffeine metabolism	Microalbuminuria	KEGG	0.019243
	Urinary albumin-to-creatinine ratio	KEGG	0.031885
Glycine serine and threonine metabolism	CKD	KEGG	0.000678
	eGFRcrea	KEGG	0.017783
Glycine and serine metabolism	CKD	SMPDB	0.007794
Glyoxylate and dicarboxylate metabolism	CKD	KEGG	0.000618
Methionine metabolism	CKD	SMPDB	0.044153
	eGFRcrea	SMPDB	0.044153
Porphyrin and chlorophyll metabolism	CKD	KEGG	0.011986
	eGFRcrea	KEGG	0.000717
	Urinary albumin-to-creatinine ratio	KEGG	0.003494
Porphyrin metabolism	CKD	SMPDB	0.038069
	eGFRcrea	SMPDB	0.003027
	Urinary albumin-to-creatinine ratio	KEGG	0.011572
Primary bile acid biosynthesis	eGFRcrea	KEGG	0.033327

### 3.4 Validation of MR results

To verify the stability of our MR analysis results, we used two new GWAS data for validation. We extracted GWAS data for the metabolites of mannose, N-acetylornithine, glycine, and bilirubin (Z, Z) in a new metabolite database. N-acetylornithine in the 486 metabolites database corresponds to N-alpha-acetylornithine and N-delta-acetylornithine in the new metabolite database. Specific MR analysis results can be seen in Table 3. It can be seen that the value of ps calculated by IVW for mannose, N-delta-acetylornithine, and glycine were 1.86E-08, 4.94E-36, and 3.13E-45, respectively, and all showed a strong causal relationship with eGFR. However, bilirubin (Z, Z) did not perform well in the new dataset with a value of *p* of 0.950196 for IVW.

**Table 3:** Results of validation MR analysis.

Exposure	Method	nSNP	B	SE	Pval	Lo_CI	Up_CI	OR	OR_Lo_CI95	OR_Up_CI95
Mannose	Inverse-variance weighted	35	−0.007835	0.001393	1.86E-08	−0.010565	−0.005104	0.992196	0.989491	0.9949087
Mannose	MR-Egger	35	−0.009861	0.003263	0.004826	−0.016257	−0.003466	0.990187	0.983875	0.9965405
Mannose	Weighted median	35	−0.006214	0.00153	4.89E-05	−0.009213	−0.003215	0.993805	0.990829	0.9967902
N-alpha-acetylornithine	Inverse-variance weighted	20	0.0066402	0.002759	0.016084	0.001233	0.012047	1.006662	1.001234	1.0121202
N-alpha-acetylornithine	MR-Egger	20	0.0022917	0.006314	0.72088	−0.010084	0.014668	1.002294	0.989966	1.0147759
N-alpha-acetylornithine	Weighted median	20	0.0012664	0.001446	0.381061	−0.001567	0.0041	1.001267	0.998434	1.0041084
N-delta-acetylornithine	Inverse-variance weighted	53	−0.005601	0.000447	4.94E-36	−0.006477	−0.004725	0.994414	0.993544	0.9952858
N-delta-acetylornithine	MR-Egger	53	−0.006901	0.000669	4.44E-14	−0.008212	−0.00559	0.993123	0.991821	0.994426
N-delta-acetylornithine	Weighted median	53	−0.006074	0.000546	8.53E-29	−0.007143	−0.005005	0.993944	0.992882	0.9950076
Glycine	Inverse-variance weighted	56	−0.009993	0.000708	3.13E-45	−0.011381	−0.008606	0.990056	0.988683	0.9914313
Glycine	MR-Egger	56	−0.014202	0.000991	4.84E-20	−0.016144	−0.012259	0.985899	0.983985	0.9878159
Glycine	Weighted median	56	−0.011306	0.000658	4.25E-66	−0.012596	−0.010015	0.988758	0.987483	0.9900348
Bilirubin (Z, Z)	Inverse-variance weighted	61	−2.87E-05	0.00046	0.950196	−0.00093	0.000873	0.999971	0.99907	1.0008733
Bilirubin (Z, Z)	MR-Egger	61	0.0006046	0.000753	0.425441	−0.000872	0.002081	1.000605	0.999129	1.0020831
Bilirubin (Z, Z)	Weighted median	61	−0.000362	0.000538	0.501106	−0.001416	0.000692	0.999638	0.998585	1.0006927

## 4 Discussion

The integration of GWAS findings of CKD, along with three indicators associated with the severity of CKD and a total of 486 blood metabolites, was conducted in this research, and then, the outcome of MR analysis, which was performed and conducted in batches, involved CKD and its associated test indicators, while the exposure consisted of 486 metabolites. In light of the high frequency of MR analysis and the large number of meaningful metabolites, Bonferroni should be used to correct and screen metabolites with high confidence, which play a key role in the occurrence and progression of CKD. Finally, four metabolites with significant causal relationships with eGFRcrea were identified: mannose, N-acetylornithine, glycine, and bilirubin (Z, Z). As mannose is causally related to all four outcomes in this study, we conclude that it is an important factor contributing to the onset and progression of CKD.

Mannose, like glucose, is also a hexose involved in human energy metabolism and other complex metabolic processes (30). Due to the fact that the GWAS data we used as a variable are only related to blood mannose content, and mannose is involved in numerous metabolic activities, it is impossible to identify the primary factors leading to the change in blood mannose content. In light of this, we discuss the following biological processes involved in mannose in relation to CKD. As a carbohydrate, mannose is a source of energy in the body. Despite not being the body’s primary energy source, mannose can influence its energy metabolism. Researchers have found that mannose-6-phosphate produced by hexokinase can affect glucose absorption by cells and then affect energy metabolism (31). Under low mannose conditions, individuals with low phosphomannose isomerase (PMI) expression often showed inhibited glucose metabolism (31). In a previous review, individuals lacking PMI were more likely to suffer from digestive tract-related diseases and to develop diseases such as liver fibrosis, diabetes, and developmental delays (32). It is speculated that the high level of mannose in the blood may inhibit the energy metabolism of the kidney, resulting in decreased kidney function and even kidney fibrosis in CKD patients.

In addition to energy metabolism, mannose-related compounds are also involved in complement activation pathways in the immune system. The mannose-binding lectin (MBL) pathway is one of the three known pathways that activate the complement system (33). In response to an interaction between MBL and the carbohydrate motif of the bacteria, mannose-binding agglutinin serine protease (MASP) cleaves C4 and C2 to produce C3 convertase C4b2b. C4b2b catalyzes the conversion of C3 into C3a and C3b and combines with C3b to form C4b2bC3b, which catalyzes the formation of C5b, thus promoting the formation of membrane attack complex (MAC) (34–36). The complement system and related immune-related products play a crucial role in glomerulonephritis progression, including IgA nephropathy (IgAN), membranous nephropathy (MN), focal segmental glomerular sclerosis (FSGS), lupus nephritis (LN), and antineutrophil cytoplasmic autoantibody-associated vasculitis (AAV) (37). According to a previous study of kidney biopsy specimens from MN patients, complement activation in the kidneys of MN patients is mainly dependent on the MBL pathway or bypass pathway, rather than the classic pathway (38). The risk of renal failure increased as C3 levels in kidney biopsy samples increased in a previous study using multivariate logistic regression (39). After all, many factors can affect the concentration level of mannose in the blood, whether it comes from oral intake or endogenous production. Whether it is from the perspective of energy metabolism or immune complement activation, the results of MR analysis can only be inferred from the existing studies, and the specific mechanism needs to be explored by subsequent experiments.

To substantiate the existing findings, we conducted additional Mendelian randomization (MR) analyses by utilizing an alternative metabolite GWAS repository as the exposure and a separate eGFR GWAS dataset as the outcome. The results reinforced the statistical robustness of mannose, N-delta-acetylornithine, and glycine, each demonstrating strong significance. However, bilirubin (Z, Z) failed to reach statistical significance in these subsequent analyses. N-delta-acetylornithine is a metabolic product synthesized by a liver- and kidney-specific N-acetyltransferase during its role in biotransformation and detoxification metabolism, involving the generation of thiol groups (40). Previous research has also identified an association between elevated circulating levels of N-delta-acetylornithine and kidney failure (41). Glycine is the simplest and most stable amino acid constituent of proteins in the human body, participating in numerous physiological and biochemical processes. However, there is no direct literature report correlating serum glycine levels with renal function in humans, offering a novel direction for future research endeavors.

MR analysis with microalbuminuria and UACR as the outcome were both enriched in the pathway for caffeine metabolism in the subsequent enrichment analysis. Furthermore, a previous study also found a causal link between coffee consumption and CKD using MR analysis, which took CKD GWAS data of CKDGen as the outcome and coffee consumption GWAS data of UK Biobank as the exposure (42). Despite using metabolite-related GWAS data as exposure, which is unrelated to the exposure data used by Kennedy et al., the same conclusion is reached, which is sufficient to demonstrate the methodological superiority of MR analysis. In addition, pathway enrichment analyses for metabolites with significant MR positive findings, using CKD, eGFRcrea, and UACR as outcomes, consistently highlighted porphyrin metabolism as a pathway of interest, regardless of whether the KEGG or the SMPDB was employed as the database. In the human body, hemochrome (ferroprotoporphyrin) is the most common porphyrin, involved in the formation of a range of proteins related to REDOX reactions, such as hemoglobin, myoglobin, cytochrome P-450 and mitochondrial cytochrome in muscle cells, and other hemoproteins in liver cells (43). A common complication of CKD is anemia, which requires iron supplementation and erythropoiesis-stimulating agents (ESAs) and even blood transfusions (17, 44). Patients with CKD will suffer from iron metabolism disorders from both the disease and subsequent treatments, and a previous study found that a significant number of dialysis patients had abnormal iron metabolisms (45). Catalytically active free iron can generate toxic reactive oxygen species (ROS), which can damage cells and their proteins (46). It is now accepted that kidney fibrosis is formed as a result of the healing response of the body (47). ROS is a recognized fibrosis-promoting molecule that promotes mesangial and fibroblast activation and tubular epithelial-to-mesenchymal cell transformation (EMT) (48). The final result is a large amount of extracellular matrix (ECM) deposition, leading to the destruction of the normal structure of the kidney and the loss of kidney function (49).

Additional metabolic pathways identified through enrichment analysis predominantly focus on primary bile acid synthesis and associated metabolite processing, which includes the metabolism of compounds such as glycine, serine, threonine, methionine, pyruvate, and dicarboxylic acids. Interestingly, glycine, serine, threonine, and methionine are all involved in the synthesis of one-carbon units, which are biologically active molecules. The primary source of one-carbon units is the carbon skeleton oxidation of amino acids, notably glycine, and both threonine and serine can be converted into glycine, thereby generating one-carbon units. The methyl group within the methionine molecule also constitutes a one-carbon unit. With the involvement of ATP, methionine is converted to S-adenosylmethionine (SAM), which is known as active methionine. Acting as a reactive methyl donor (50, 51). One-carbon units predominantly contribute to cellular proliferation through the biosynthesis of purines and deoxythymidine monophosphate (dTMP) (52). The proliferation and activation of early immune cells consequently affect renal function, while the excessive proliferation of fibroblasts during the chronic progression phase is a common pathological hallmark of chronic kidney disease (53, 54). Research on the association between bile acid synthesis-related pathways and chronic kidney disease is limited, and bilirubin (Z, Z) lacked statistical significance in subsequent validation datasets.

However, our study has some limitations. The condition selection for SNP screening is relatively loose in order to provide sufficient instrumental variables for subsequent MR analysis, although we eliminate weaker IVs by using *F* > 10. Additionally, this study made use of a large number of two-sample MR analyses, which are susceptible to multiple test errors, although we corrected our results with Bonferroni correction. Finally, it cannot be denied that MR analysis is an effective tool for the study of etiology. However, metabolites that have a causal relationship with CKD screened in this study lack further experimental verification and exploration of their specific mechanisms, which also needs to be further improved in this study.

## 5 Conclusion

As a result of MR analysis, 78 metabolites were found to have causal relationships with CKD or its related indicators. After Bonferroni correction, mannose, N-acetylornithine, glycine, and bilirubin (Z, Z) remained robust. Eight metabolic pathways were identified after enrichment analysis of metabolic pathways related to CKD occurrence and progression. It is these metabolites and their subsequent metabolic pathways that can be used to identify high-risk patients in the early stages of CKD and take preventative measures or to prevent the progression of CKD later on. In addition, it provides direction for further research on the etiology and pathogenesis of CKD.

## Data availability statement

Publicly available datasets were analyzed in this study. This data can be found at: https://gwas.mrcieu.ac.uk/ and https://www.metaboanalyst.ca.

## Ethics statement

Ethical approval was not required for the study involving humans in accordance with the local legislation and institutional requirements. Written informed consent to participate in this study was not required from the participants or the participants' legal guardians/next of kin in accordance with the national legislation and the institutional requirements.

## Author contributions

YY: Conceptualization, Data curation, Formal analysis, Writing – original draft, Investigation, Methodology, Resources, Software, Supervision, Visualization. CS: Conceptualization, Data curation, Formal analysis, Investigation, Methodology, Resources, Software, Supervision, Visualization, Writing – original draft. QH: Conceptualization, Data curation, Formal analysis, Investigation, Methodology, Resources, Software, Supervision, Validation, Visualization, Writing – original draft, Writing – review & editing. CC: Methodology, Validation, Writing – review & editing. ZW: Supervision, Validation, Writing – review & editing. ZHu: Supervision, Validation, Writing – review & editing. HC: Supervision, Validation, Writing – review & editing. LS: Supervision, Validation, Writing – review & editing. SF: Supervision, Validation, Writing – review & editing. JT: Supervision, Validation, Writing – review & editing. ZHa: Supervision, Validation, Writing – review & editing. RT: Supervision, Validation, Writing – review & editing. MG: Funding acquisition, Project administration, Resources, Writing – review & editing. XJ: Conceptualization, Data curation, Formal analysis, Funding acquisition, Investigation, Methodology, Project administration, Resources, Software, Supervision, Validation, Visualization, Writing – review & editing.

## Footnotes

^1^ https://metabolomics.helmholtz-muenchen.de/gwas/

^2^ https://gwas.mrcieu.ac.uk/

^3^ https://www.metaboanalyst.ca/

## Supplementary material

SUPPLEMENTARY TABLE 1 IVs for MR Analysis. Notes: IVs: Instrumental Variables, MR Analysis: Mendelian Randomization Analysis.

SUPPLEMENTARY TABLE 2 Deleted SNPs associated with confounding factors.

SUPPLEMENTARY TABLE 3 All MR Analysis results of 5 MR Analysis methods with the same direction.

SUPPLEMENTARY TABLE 4 Sensitivity analysis results of metabolite which was meaningful for MR Analysis.

SUPPLEMENTARY FIGURE 1 Three underlying assumptions of MR analysis.

SUPPLEMENTARY FIGURE 2 The underlying assumptions of MR analysis.
