# Supplementary figures and images for: Causal effects of human serum metabolites on occurrence and progress indicators of chronic kidney disease: a two-sample Mendelian randomization study
Source: Front Nutr. 2024 Jan 8;10:1274078. doi: 10.3389/fnut.2023.1274078 (PMC10800733; doi:10.3389/fnut.2023.1274078)

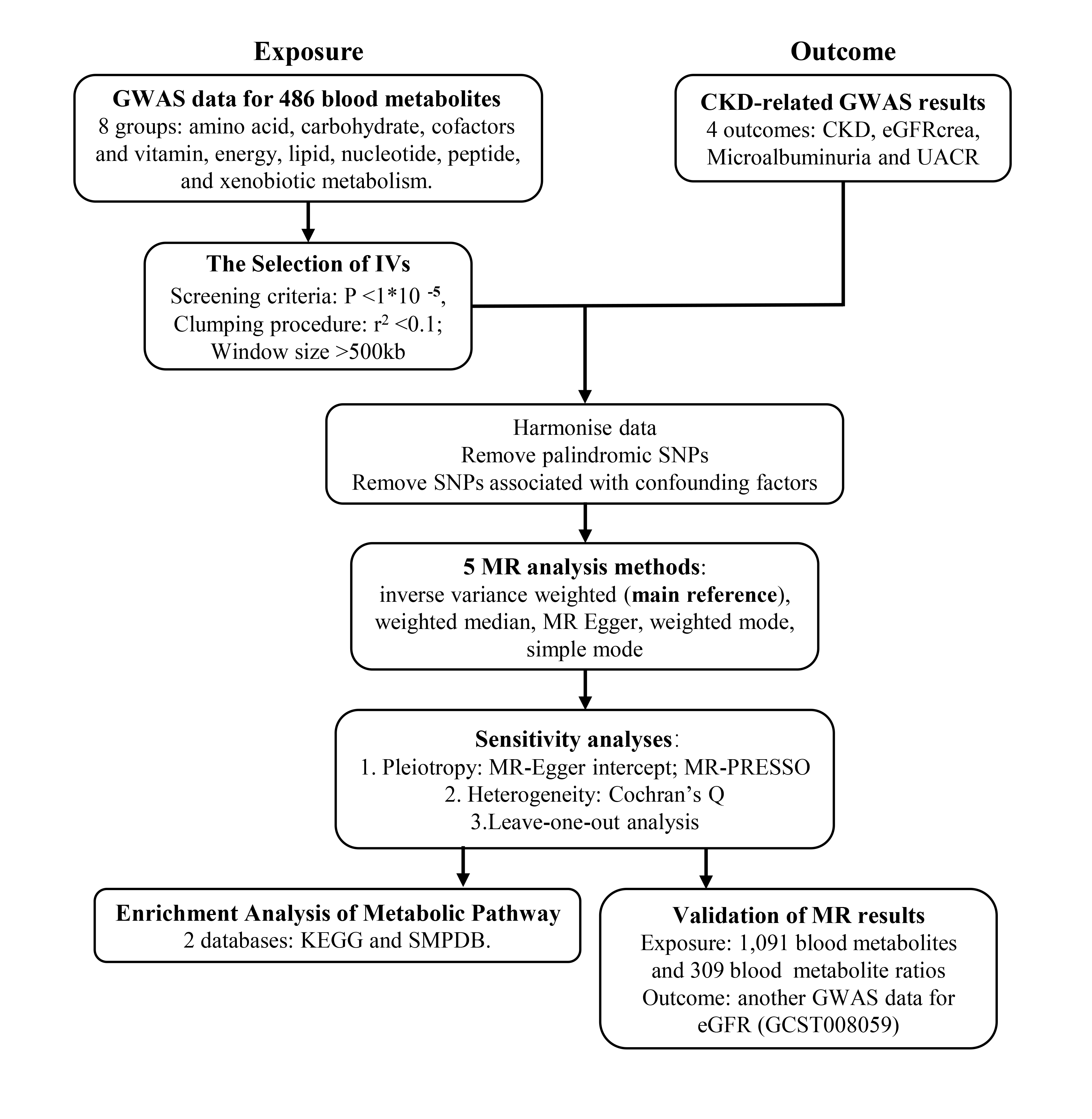

Supplement: SUPPLEMENTARY FIGURE 1 — Three underlying assumptions of MR analysis. [file Image_1.tif]

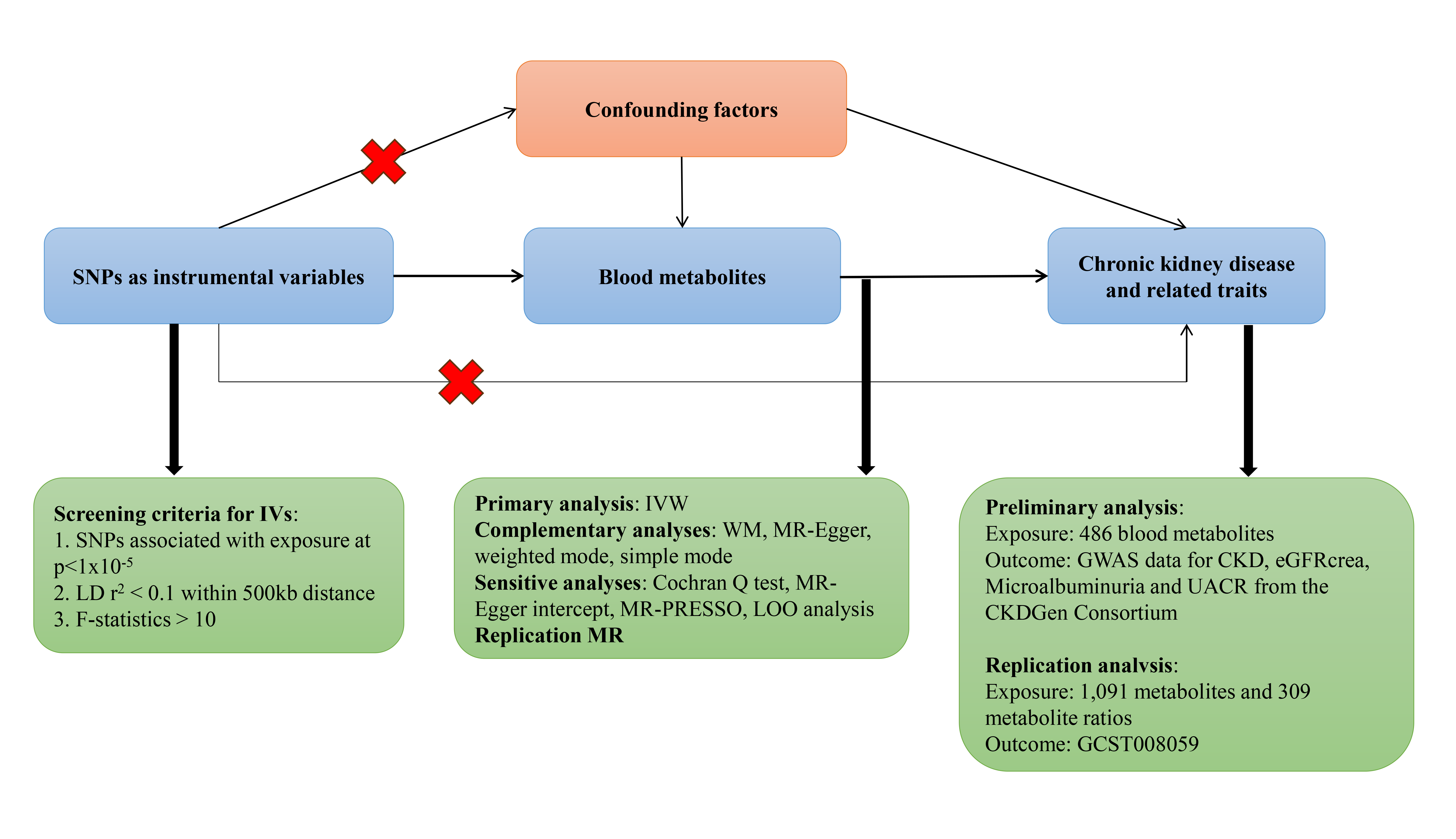

Supplement: SUPPLEMENTARY FIGURE 2 — The underlying assumptions of MR analysis. [file Image_2.tif]
